# Zerumbone Exhibits Antiphotoaging and Dermatoprotective Properties in Ultraviolet A-Irradiated Human Skin Fibroblast Cells via the Activation of Nrf2/ARE Defensive Pathway

**DOI:** 10.1155/2019/4098674

**Published:** 2019-11-14

**Authors:** You-Cheng Hseu, Chih-Ting Chang, Yugandhar Vudhya Gowrisankar, Xuan-Zao Chen, Hui-Chang Lin, Hung-Rong Yen, Hsin-Ling Yang

**Affiliations:** ^1^Department of Cosmeceutics, College of Biopharmaceutical and Food Sciences, China Medical University, Taichung 40402, Taiwan; ^2^Department of Health and Nutrition Biotechnology, Asia University, Taichung 41354, Taiwan; ^3^Chinese Medicine Research Center, China Medical University, Taichung 40402, Taiwan; ^4^Research Center of Chinese Herbal Medicine, China Medical University, Taichung 40402, Taiwan; ^5^Department of Biotechnology, Asia University, Taichung 41354, Taiwan; ^6^School of Pharmacy, China Medical University, Taichung 40402, Taiwan; ^7^Department of Chinese Medicine, China Medical University Hospital, Taichung 404, Taiwan; ^8^Research Center for Traditional Chinese Medicine, Department of Medical Research, China Medical University Hospital, Taichung 404, Taiwan; ^9^School of Chinese Medicine, College of Chinese Medicine, China Medical University, Taichung 404, Taiwan; ^10^Institute of Nutrition, College of Biopharmaceutical and Food Sciences, China Medical University, Taichung 40402, Taiwan

## Abstract

Ultraviolet A (UVA) irradiation (320-400 nm range) triggers deleterious consequences in skin cell microenvironment leading to skin damage, photoaging (premature skin aging), and cancer. The accumulation of intracellular reactive oxygen species (ROS) plays a key role in this effect. With rapid progress in cosmetic health and quality of life, use of safe and highly effective phytochemicals has become a need of the hour. Zerumbone (ZER), a natural sesquiterpene, from *Zingiber zerumbet* rhizomes is well-known for its beneficial effects. We investigated the antiphotoaging and dermatoprotective efficacies of ZER (2-8 *μ*M) against UVA irradiation (3 J/cm^2^) and elucidated the underlying molecular mechanisms in human skin fibroblast (HSF) cells. ZER treatment prior to low dose of UVA exposure increased cell viability. UVA-induced ROS generation was remarkably suppressed by ZER with parallel inhibition of MMP-1 activation and collagen III degradation. This was due to the inhibition of AP-1 (c-Fos and c-Jun) translocation. Furthermore, ZER alleviated UVA-induced SA-*β*-galactosidase activity. Dose- or time-dependent increase of antioxidant genes, HO-1 and *γ*-GCLC by ZER, was associated with increased expression and nuclear accumulation of Nrf2 as well as decreased cytosolic Keap-1 expressions. Altered luciferase activity of ARE could explain the significance of Nrf2/ARE pathway underlying the dermatoprotective properties of ZER. Pharmacological inhibition of various signaling pathways suppressed nuclear Nrf2 activation in HSF cells confirming that Nrf2 translocation was mediated by ERK, JNK, PI3K/AKT, PKC, AMPK, casein kinase II, and ROS signaling pathways. Moreover, increased basal ROS levels and Nrf2 translocation seem crucial in ZER-mediated Nrf2/ARE signaling pathway. This was also evidenced from Nrf2 knocked-out studies in which ZER was not able to suppress the UVA-induced ROS generation in the absence of Nrf2. This study concluded that in the treatment of UVA-induced premature skin aging, ZER may consider as a desirable food supplement for skin protection and/or preparation of skin care products.

## 1. Introduction

Skin aging is a cascade of complex biological events driven by intrinsic/endogenous (genetically programmed hormonal, cellular, and metabolic processes) and extrinsic/exogenous (ionizing radiation, toxic chemicals, pollutants, and chronic light exposure) factors cumulatively lead to progressive alterations in skin physiology and appearance [1]. Human skin is constantly exposed to UV irradiation present in sunlight. Depending on the wavelength and energy levels, UV light may be of the following three types: A, B, or C. Among these, UVA (315-400 nm) is the major constituent with high penetration capacity [2]. It penetrates deep into the dermis compartment of skin and causes endogenous “photosensitization-” mediated “oxidative stress” in the skin cells leading to early signs of “photoaging” and cutaneous photodamage [3, 4]. Photoaging can be attributed to extrinsic skin aging that includes sagging, laxity, patchy/mottled pigmentation, increased wrinkling, and dryness [5, 6].

In the dermal compartment, UVA triggers the generation of large concentrations of ROS species, viz hydrogen peroxide (H_2_O_2_), free radicals, singlet oxygen, and superoxide. Due to their high activity, they interact with each other and induce a cascade of photodamaging events [7, 8]. Rijken and Bruijnzeel reported that alterations in extracellular matrix (ECM) and connective tissue damage were most likely caused by excessive matrix degradation mediated by UVA-induced matrix metalloproteinase (MMP) enzymes that play a pivotal role in collagen degradation [9]. MMP-1 is such a collagenase enzyme, secreted from keratinocytes and skin fibroblast cells, that hydrolyzes collagen type III protein and induces hydrolysis, destruction, and restructuring of ECM that lead to clinical manifestation of skin aging [10–13]. Previous reports have suggested that the activation of mitogen-activated protein kinase (MAPK) signal transduction pathway and upregulation in transcription factor activator protein- (AP-) 1 levels are mediating the expression of MMPs [14–16].

Skin cells are well equipped with an elaborate antioxidant system that neutralizes oxidative stress posed by UVA irradiation and maintains cellular homeostasis [17]. The Keap-1-Nrf2 [Kelch-like ECH-associated protein 1-nuclear factor (erythroid-derived 2)-like 2] regulatory complex present in the cytoplasm plays a central role in protecting cells against oxidative, xenobiotic stresses and maintains cellular homeostasis. Nrf2 is a redox-sensitive basic leucine zipper protein (bZIP) and a major transcriptional enhancer known to be involved in the gene expression of distinct phase II detoxifying enzymes and cytoprotective protein coding genes [18, 19]. Itoh et al. explained that Keap-1 via its thiol groups interacts with oxidative inducers and triggers the release of Nrf2 from Nrf2-Keap-1 complex in the cytoplasm that translocate to the nucleus for further activation of ARE, a cis-regulatory DNA sequence located in the promoter region [20] of phase II detoxifying enzymes leading to the expression of HO-1 or *γ*-GCLC proteins [21, 22]. These proteins are involved in antioxidant defensive mechanism through their anti-inflammatory, antiapoptotic, and antiproliferative properties in various cells and tissue types [23–26]. Therefore, Keap-1-Nrf2 system acts like a double-edged sword and Nrf2 is considered a master switch for the transcriptional activation of various genes [27, 28].

Interventional studies showed that the administration of plant-derived natural nutritional supplements could protect the skin from the deleterious effects of UV irradiation [29]. “Zerumbone (ZER),” is one such key compound isolated from the fresh rhizome of *Zingiber zerumbet* Smith, belongs to *Zingiberaceae* family. It is widely distributed in Southeast Asia and cultivated for both culinary and medical purposes [30]. ZER contains an 11-membered monocyclic sesquiterpene that has remarkable pharmacological profile with medicinal importance [31]. ZER was reported to exhibit a Nrf2/ARE-dependent detoxification pathway [22]. The *α*,*β*-unsaturated carbonyl moiety present in this compound exhibited various biological activities [32], such as the inhibition of tumor cell growth [33], induction of differentiation [34], apoptosis [35], insulin-like growth factor-I and Waf-1 [36], heat shock protein [37], glutathione *S*-transferase activity [38], and cytoprotective activity [39]. However, the structural analogues of ZER, *α*-humulene and 8-hydroxy-*α*-humulene, lack the *α*,*β*-unsaturated carbonyl moiety needed to activate the Nrf2 pathway [40]. In our previous study, we showed that Nrf2 activation was pivotal for ZER to exhibit its beneficial effects in human skin keratinocytes [41]. Taken together, this study delineated the molecular mechanisms that underlie the curative and antiphotoaging efficacy of ZER against UVA radiation-induced photodamage and photoaging on human skin fibroblast cells.

## 2. Materials and Method

### 2.1. Reagents and Test Substances

3-[4,5-Dimethyl-2-yl]-2,5-diphenyl tetrazolium bromide (MTT), zerumbone (ZER) (purity ≥ 98%), 2′,7′-dichlorofluorescin-diacetate (DCFH_2_-DA), LY294002 (PI_3_K/AKT inhibitor), and *N*-acetylcysteine (NAC) were obtained from Sigma-Aldrich (St. Louis, MO). SB203580 (p38 MAPK inhibitor), PD98059 (ERK inhibitor), GF109203X (PKC inhibitor), and SP600125 (JNK inhibitor) were purchased from Calbiochem (La Jolla, CA). Compound C (AMPK inhibitor) and CKII (casein kinase II inhibitor) were obtained from Merck & Co., Inc. (Darmstadt, Germany). The following antibodies were purchased from their respective companies: p-c-Fos, p-c-Jun, Nrf2, Keap-1, *β*-actin [Santa Cruz Biotechnology Inc. (Heidelberg, Germany)]; histone [Cell Signaling Technology (Beverly, MA, USA)]; anti-HO-1 and anti-*γ*-GCLC [Gene Tex Inc. (San Antonio, TX, USA)]; and MMP-1, collagen type III [Abcam Inc. (Cambridge, MA, USA)]. All other chemicals, tissue culture, and common laboratory supplies were either purchased from GIBCO BRL (Grand Island, NY, USA) or Merck & Co., Inc. (Darmstadt, Germany) or Sigma-Aldrich (St. Louis, MO).

### 2.2. Cell Culture

Human skin fibroblast (HSF) cell line was obtained from BCRC (Bioresource Collection and Research Center, Hsinchu, Taiwan). These cells were expanded and maintained in 25 cm^2^ tissue culture flasks with DMEM containing 10% fetal bovine serum (FBS), 10,000 *μ*g/mL streptomycin, and 10,000 I.U./mL penicillin at 37°C in a humidified incubator (5% CO_2_ and 95% air).

### 2.3. ZER Pretreatment and UVA Irradiation

HSF cells were treated with various concentrations of ZER (2, 4, or 8 *μ*M) or vehicle (0.1% DMSO) for 24 h or 2 h. After treatments, cells were washed with phosphate-buffered saline (PBS) and resuspended in fresh phenol red-free DMEM containing 10% FBS. In our recent study, 3 J/cm^2^ UVA dosage indicated less cytotoxicity (~90% cell viability) [41]. As such, we applied the same dosage throughout this study. Using an UVI link CL-508L (UVItec, Cambridge, UK), HSF cells were irradiated with 3 J/cm^2^ UVA for about 27 min (*λ*_max_ 365 nm; no detectable emission below 320 nm). After irradiation, cells were allowed to be incubated for 2 h (for the expression of proteins) before proceeding to other experiments. A stock concentration of 50 mM ZER was prepared with DMSO and stored at -20°C for further analysis.

### 2.4. Cell Viability (MTT) Assay

The effect of ZER-mediated cell viability in UVA-irradiated HSF cells was measured by MTT colorimetric assay. Approximately, 0.1 million HSF cells/well in a 12-well plate were treated with 0, 2, 4, or 8 *μ*M concentrations of ZER for 24 h and then exposed to UVA (3 J/cm^2^). These cells were washed with PBS and incubated for 2 h in 400 *μ*L of 0.5 mg/mL MTT in PBS. To dissolve the MTT formazan crystals, we replaced the supernatant with 400 *μ*L of isopropanol. The intensity of color developed due to dissolved MTT formazan was measured with an ELISA microplate reader (Bio-Tek Instruments, Winooski, VT, USA) at 570 nm. Data were represented as percentage of viable cells as compared to vehicle-treated control cells, which were arbitrarily assigned a viability value of 100%.

### 2.5. Preparation of Cell Extracts from HSF Cells

All protein extraction procedures were conducted on ice-cold conditions and reagents. HSF cells were pretreated with different concentrations of ZER (2- 8 *μ*M) for the indicated time and were exposed to UVA. Subsequently, the cells were detached and washed with cold PBS and solubilized in lysis buffer (10 mM HEPES—pH 8.0, 0.1 mM EDTA, 10 mM KCl, 100 *μ*M EGTA, 1 mM DTT, 500 *μ*M PMSF, 2.0 *μ*g/mL leupeptin, 2.0 *μ*g/mL aprotinin, and 500 *μ*g/mL benzamidine) for 15 min. This suspension was centrifuged at 15,000 g for 20 min at 4°C, and the protein content in total, cytoplasmic, and nuclear fractions was determined using a Bio-Rad protein assay reagent (Bio-Rad, Hercules, CA, USA), with bovine serum albumin as the standard as described previously [42]. These protein samples were preserved in −80°C freezer until further use.

### 2.6. Western Blot Method

Equal concentrations (60 *μ*g) of solubilized proteins were separated in 8-15% polyacrylamide gradient gels (PAGE) and transferred onto polyvinylidene difluoride (PVDF) membranes. Nonspecific binding to the membranes was prevented by the incubation with 5% Blotto (5% nonfat dry milk and 1% Tween-20 in PBS) for at least 1 h at room temperature. Membranes were then incubated with various primary antibodies overnight at 4°C. On the following day, membranes were washed and further incubated for 2 h in the presence of horseradish peroxidase- (HRP-) conjugated goat anti-rabbit or anti-mouse secondary antibodies at room temperature. The immune reactive protein bands were visualized using Super Signal West Pico chemiluminescence substrate (Thermo Scientific Inc., Rockford, IL, USA), and images were taken from Image Quant™ LAS 4000 mini (Fujifilm) system. Protein loading was normalized with a *β*-actin (Santa Cruz Biotechnology Inc., Heidelberg, Germany) or histone proteins (Cell Signaling Technology, Beverly, MA, USA)

### 2.7. DCFH_2_-DA Measurement of Intracellular ROS Levels

2′,7′-Dichlorodihydrofluorecein diacetate (DCFH_2_-DA) is a membrane permeable fluorescent dye that gets hydrolyzed by intracellular esterase. Due to intracellular ROS, DCFH_2_-DA oxidized to a fluorescent product dichlorofluorescein (DCF) gets trapped inside the cells and emits fluorescent signals [43]. To determine the effect of UVA radiation on ROS levels, HSF cells were pretreated with ZER (2-8 *μ*M, 24 h) and irradiated with UVA (3 J/cm^2^). Cells were washed with PBS and incubated in the presence of 10 *μ*M DCFH_2_-DA (diluted in fresh DMEM) for 30 min at 37°C. Using an Olympus 1 × 71 fluorescence microscope (200x magnification), the fluorescence intensity emitted by fluorescence-stained cells was measured and analyzed using analySIS LS 5.0 soft imaging solutions (Olympus Imaging America Inc., Corporate Parkway Centre Valley, PA, USA). Data were represented as fold over change as compared to control DCF values.

### 2.8. Senescence-Associated *β*-Galactosidase (SA-*β*-Gal) Staining

The SA-*β*-Gal staining method was used to measure the cellular senescence activity [44]. In the current study, we measured SA-*β*-gal activity in HSF cells using a commercially available cellular senescence assay kit (Millipore, USA) with the prescribed protocol. ZER-pretreated (2-8 *μ*M, 24 h) and UVA-irradiated (3 J/cm^2^) HSF cells were washed with PBS and fixed using glutaraldehyde solution. These cells were incubated with fresh SA-*β*-gal staining solution for overnight at 37°C. On the following day, using a reverse-phase microscope, images of SA-*β*-Gal-positive cells were taken at a total magnification of 200x.

### 2.9. Immunofluorescence

ZER-induced subcellular localization of antioxidant protein expression was measured using a immunofluorescence method. ZER-pretreated (2-8 *μ*M for the indicated time) and/or UVA-irradiated (3 J/cm^2^) HSF cells were washed and fixed with 4% paraformaldehyde, followed by permeabilized with 0.1% Triton X-100 for 10 min. These HSF cells were blocked (10% FBS) and incubated in the presence of different primary antibodies (anti-p-c-Fos or anti-p-c-Jun or anti-Nrf2, prepared in 1.5% FBS) for overnight. On the subsequent day, cells were washed and labeled with FITC [(fluorescein isothiocyanate)-conjugated (488 nm)] secondary antibody for 1 h in 6% bovine serum albumin solution. These cells were counterstained with 4′,6′-diamino-2-phenylindole (DAPI) for 5 min and examined under fluorescence microscope. Images were captured at a total magnification of 200x.

### 2.10. Nrf2 Small Interference RNA (siRNA) Transfection in HSF Cells

0.5 million HSF cells were cultured and allowed to reach 40-60% confluence. On the following day, the culture medium was replaced and cells were transfected using Lipofectamine RNAiMAX kit (Invitrogen, Carlsbad, CA, USA) to knock down Nrf2. The manufacturer's protocol for transfection was followed as described. After transfection, HSF cells were treated with ZER for 2 h, and the expression of Nrf2 and HO-1 protein levels in these cells was determined using the western blot method described previously in this article.

### 2.11. Statistical Analysis

All data were analyzed using analysis of variance (ANOVA) followed by Dunnett's test for pairwise comparisons using Sigma Plot 10.0 statistical software. The data were represented as mean ± standard deviation (mean ± SD) of three or more experiments. The criterion for statistical significance was ∗*p* < 0.05, ∗∗*p* < 0.01, ∗∗∗*p* < 0.001 compared with untreated control cells; ^#^*p* < 0.05, ^##^*p* < 0.01, ^###^*p* < 0.001 compared with UVA-irradiated (or) ZER-treated cells.

## 3. Results

### 3.1. UVA-Induced Cell Death Was Suppressed in ZER-Pretreated HSF Cells

**Figure 1:**
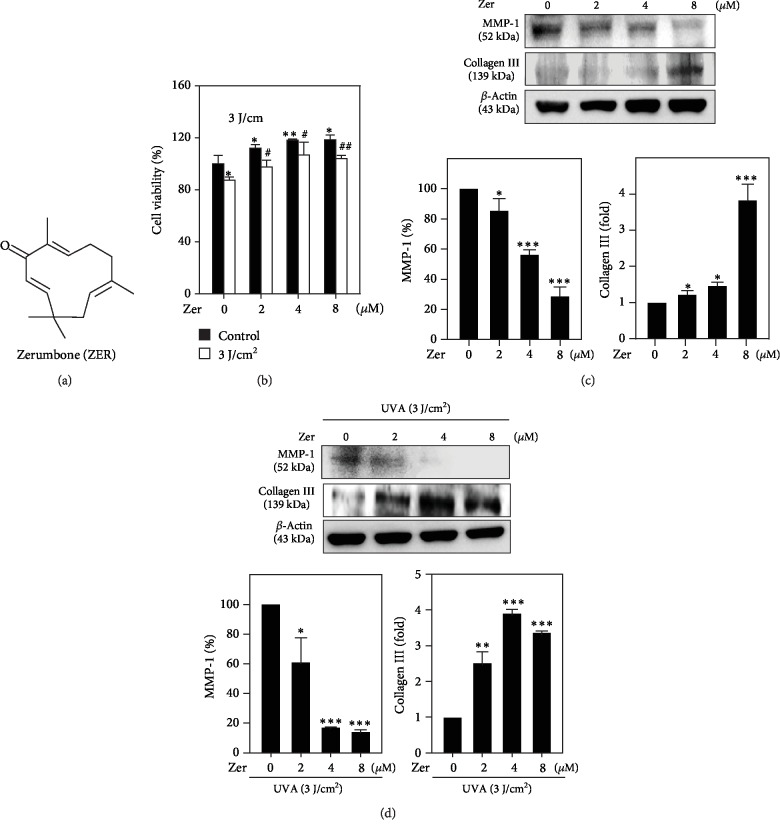
Effect of ZER on cell viability and altered MMP-1 and collagen III expressions in UVA-irradiated HSF cells. (a) Chemical structure of zerumbone (ZER). (b) Cells were pretreated with ZER (0, 2, 4, or 8 *μ*M for 24 h) followed by UVA irradiation (3 J/cm^2^ for 27 min). 24 h after UVA exposure, the percentage of cell viability was measured by MTT colorimetric assay as described. The formula used to calculate the percentage of viable cells was (*A*_570_ of treated cells/*A*_570_ of untreated cells) × 100. (c, d) HSF cells were pretreated with ZER (0, 2, 4, or 8 *μ*M for 24 h), and the expression of MMP-1 and collagen III proteins in the absence (c) or presence (d) of UVA radiation was measured using the western blot method. Results were presented as mean ± SD of three or more assays. ∗*p* < 0.05, ∗∗*p* < 0.01, ∗∗∗*p* < 0.001 compared to untreated control cells; ^#^*p* < 0.05, ^##^*p* < 0.01 compared to UVA-irradiated cells.

The *in vitro* cell viability efficiency of ZER (Figure 1(a)) against UVA-irradiated HSF cells was tested by MTT colorimetric assay. Data showed that compared to the untreated cells, UVA radiation decreased HSF cell viability by 10%. However, ZER pretreatment dose-dependently protected the cells to undergo UVA radiation-induced cell death with maximum cell viability and proliferations were observed at 8 *μ*M ZER concentration (Figure 1(b)).

### 3.2. ZER Attenuated UVA-Induced MMP-1 Expression and Downregulated the Collagen III Degradation in HSF Cells

MMP-1 activation and collagen III degradation are two principle events associated with UVA radiation-induced premature skin aging process [45, 46]. Therefore, we determined the changes in MMP-1 and collagen III protein expression levels in ZER-pretreated (2-8 *μ*M, 24 h) and UVA-irradiated (3 J/cm^2^ for 27 min) HSF cells. As represented in Figure 1(c), in the absence of UVA exposure, ZER pretreatment dose-dependently and significantly downregulated MMP-1 expression, but upregulated collagen III levels, whereas MMP-1 expression was significantly suppressed due to ZER pretreatment in UVA-irradiated HSF cells leading to sustained expression of collagen III protein levels (Figure 1(d)). This data explains the anticollagenolytic activity of ZER in UVA-mediated skin damage and premature skin aging.

### 3.3. ZER Decreased UVA-Induced Intracellular ROS Accumulation in HSF Cells

**Figure 2:**
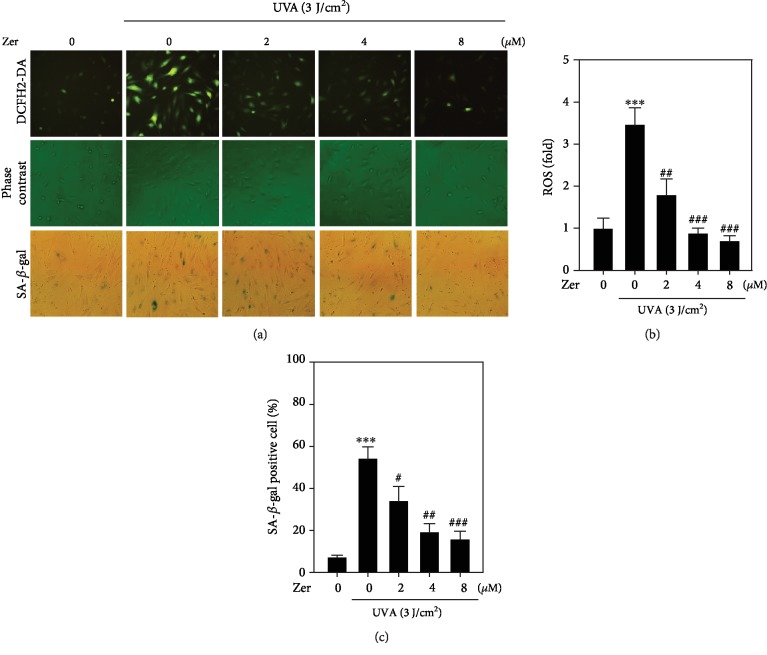
Effect of ZER on intracellular ROS levels and SA-*β*-gal activity in UVA-irradiated HSF cells, (a) Cells were pretreated with ZER (0, 2, 4, or 8 *μ*M for 24 h) followed by irradiated with UVA in the absence or presence of 3 J/cm^2^ UVA. The accumulation of UVA-induced ROS and SA-*β*-gal-positive cells was measured using fluorescence microscopy (200x magnification) as described. (b) The data was represented as fold change over control intracellular ROS levels. (c) The data was reported as fold increase over control SA-*β*-gal-positive cells. Results from three or more experiments were presented as mean ± SD, and the statistical significance was considered as ∗∗∗*p* < 0.001 compared to untreated control cells; ^#^*p* < 0.05, ^##^*p* < 0.01, ^###^*p* < 0.001 compared to UVA-irradiated cells.

Previous studies have demonstrated the link between increased accumulation of intracellular ROS with UVA radiation insults that lead to photoaging and skin cancer [42, 47]. To see if ZER has antioxidant abilities to alleviate the UVA-mediated ROS production in HSF cells, DCFH_2_-DA fluorescence staining method was used to measure the fluorescence intensity emitted by DCF-positive cells that were pretreated with different concentrations of ZER (2-8 *μ*M) and irradiated with UVA. Compared to the control cells, UVA radiation alone trigged significant upregulation in the accumulation of ROS in HSF cells. However, this effect was dramatically alleviated in ZER-pretreated HSF cells in a concentration-dependent manner (Figures 2(a) and 2(b)). Hence, ZER exhibited antioxidative activity in UVA-irradiated HSF cells.

### 3.4. ZER Prevented UVA-Irradiated HSF Cells to Undergo Premature Skin Cell Aging

Cellular senescence is a detrimental consequence of radiation-induced stress in dermal cells [3]. Senescence-associated *β*-galactosidase is a key biomarker to determine the senescence activity in cells that were exposed to stress stimuli [44]. Using a SA-*β*-gal kit, we measured the radiation-induced cellular senescence activity in HSF cells. Our data showed that there was approximately 55% SA-*β*-gal-positive cell activity in UVA alone-exposed HSF cells (Figures 2(a) and 2(c)). However, this effect was significantly attenuated in ZER-pretreated cells in a dose-dependent manner. 8 *μ*M ZER showed maximum antisenescence activity in UVA-irradiated HSF cells (Figures 2(a) and 2(c)).

### 3.5. ZER Inhibited c-Fos and c-Jun Phosphorylations in UVA-Irradiated HSF Cells

**Figure 3:**
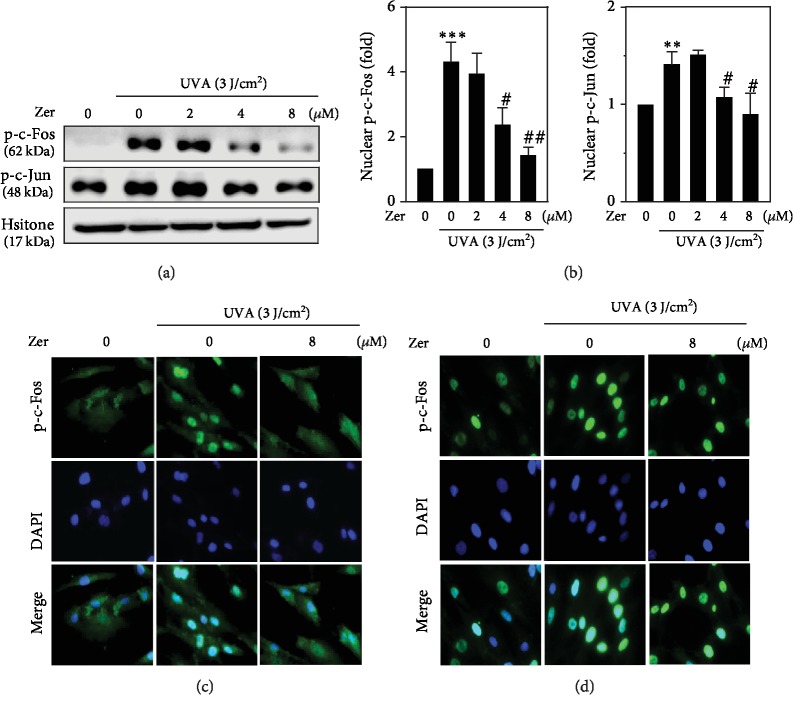
Effect of ZER on activator protein- (AP-) 1-associated protein expression in UVA-irradiated HSF cells. Cells were pretreated with ZER (0, 2, 4, or 8 *μ*M for 24 h) followed by irradiation in the absence or presence of 3 J/cm^2^ UVA for the indicated time. After irradiation, cells were allowed to incubate for 2 h and the subsequent experiments were performed. (a, b) The expression of p-c-Fos and p-c-Jun proteins in the absence or presence of UVA at various concentrations of ZER was measured using western blot method. Also, an immunofluorescence staining was also performed to measure the alterations in p-c-Fos (c) and p-c-Jun (d) expressions as described in the methodology. Results from three or more experiments were presented as mean ± SD, and the statistical significance was considered as ∗∗*p* < 0.01, ∗∗∗*p* < 0.001 compared to untreated control cells; ^#^*p* < 0.05, ^##^*p* < 0.01 compared to UVA-irradiated cells.

Activator protein- (AP-) 1 is a heterodimer transcription factor composed of c-Fos and c-Jun families of proteins that regulate the expression of genes in response to a variety of stimuli. Previous reports suggested that activation of c-Fos and c-Jun is key in UVA-induced photoaging process [47, 48]. Therefore, we determined the alterations in nuclear localization of phosphorylated c-Fos and c-Jun proteins in ZER-pretreated HSF cells that were exposed to UVA stress. Results from our study showed that UVA radiation alone tends to elevate the phosphorylations of c-Fos and c-Jun proteins in HSF cells. However, this effect was dose-dependently and significantly downregulated due to ZER pretreatment (Figures 3(a) and 3(b)). Later, we conducted immunofluorescence staining experiments to detect the nuclear localization of p-c-Fos and p-c-Jun in HSF cells. Immunofluorescence images made it clear that 8 *μ*M ZER pretreatment was able to control the UVA-induced nuclear accumulation of p-c-Fos and p-c-Jun in fibroblasts (Figures 3(c) and 3(d)).

### 3.6. Effect of ZER on Nuclear Translocation of Nrf2 in HSF Cells

**Figure 4:**
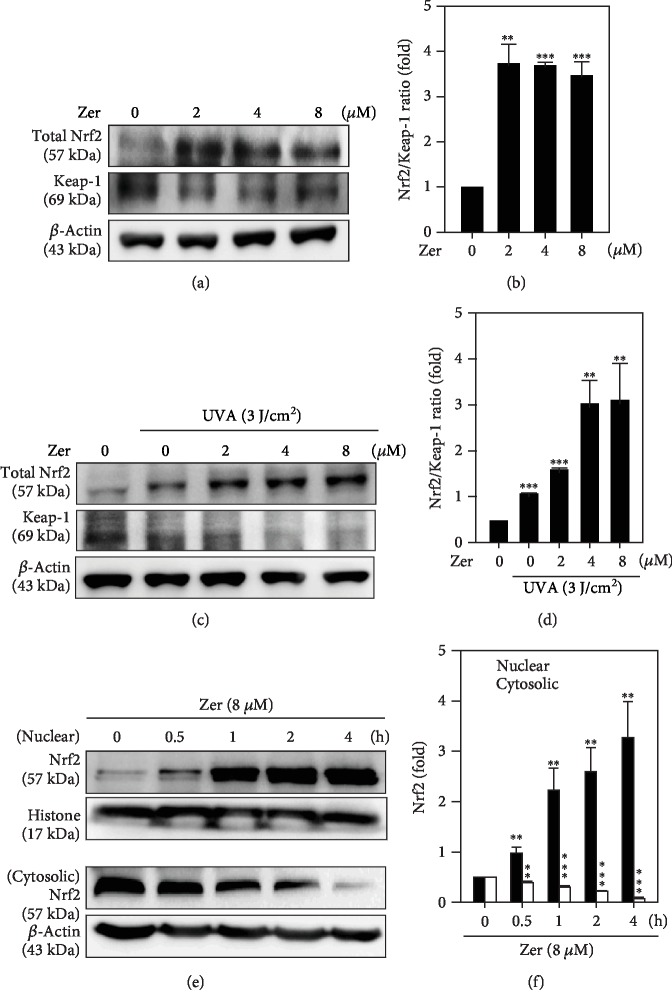
Effect of ZER on the nuclear translocation, activation of Nrf2, and its associated proteins in UVA-irradiated HSF cells: ZER pretreated (2-8 *μ*M for 2 h) HSF cells were irradiated in the presence (a, b) or presence (c, d) of 3 J/cm^2^ UVA (for the indicated time) were subjected to western blot for the measurement of total Nrf2 and Keap-1 protein expressions. The effect of ZER on this pattern was represented as fold change of Nrf2/Keap-1 ratio over the control values. This ratio determines the dissociation pattern of Nrf2 from Keap-1 in the cytoplasm. (e, f) HSF cells were treated with 8 *μ*M of ZER at different time points (0, 0.5, 1, 2, or 4 h), and the expression of cytosolic, nuclear Nrf2 protein fractions was determined using western blot method. *β*-Actin and histone proteins were used as internal controls for cytosolic and nuclear Nrf2, respectively. Results from three or more experiments were presented as mean ± SD, and the statistical significance was considered as ∗∗*p* < 0.01, ∗∗∗*p* < 0.001 compared to untreated control cells.

In the cytoplasm, Nrf2 is a redox-sensitive transcription factor associated with its inhibitor protein Keap-1 in a complex form. However, sequestration of Keap-1 protein from this complex leads to Nrf2 to translocate in the nucleus for the expression of antioxidant proteins [49]. In this study, we observed that ZER treatment (2-8 *μ*M, 2 h) dose-dependently increased the total Nrf2 protein levels in UVA nonexposed (Figures 4(a) and 4(b)) or UVA-exposed (Figures 4(c) and 4(d)) HSF cells. Degradation of Keap-1, in the presence of ZER, is confirming that Nrf2 activation was mediated via the sequestration of Keap-1 in cytosol (Figures 4(a)–4(d)). The effect of time on ZER to mediate nuclear translocation of Nrf2 was measured. Western blot data showed that nuclear Nrf2 was observed from 0.5 h time point and was gradually increased up to 4 h. This was also corroborated with proportional decrease in the cytosolic Nrf2 levels in HSF cells (Figures 4(e) and 4(f)). This effect was measured to be statistically significant. This data clearly inferred the ZER-mediated dissociation of Nrf2-Keap-1 complex in cytoplasm and favoring the translocation of Nrf2 to nucleus to mediate its antioxidative downstream effects.

### 3.7. ZER Upregulated *γ*-GCLC and HO-1 Protein Expressions via the Transcriptional Activation of Nrf2 in HSF Cells

**Figure 5:**
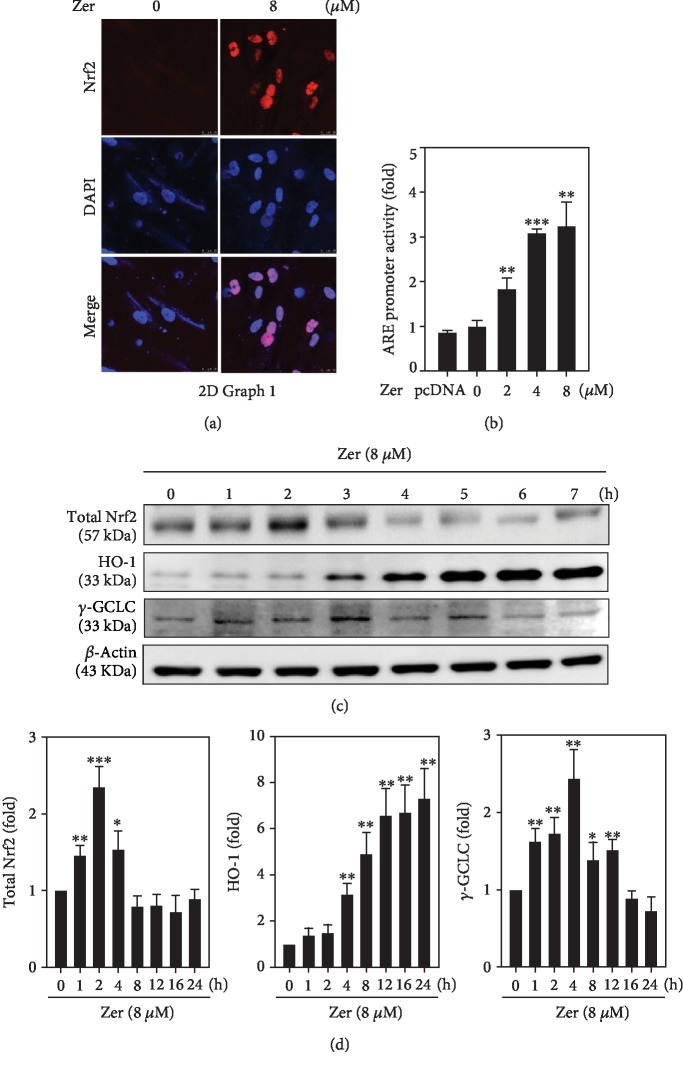
Effect of ZER on ARE promoter activation and subsequent expression of HO-1 and *γ*-GCLC proteins in HSF cells. (a) Cells were treated with ZER (8 *μ*M for 2 h) and subcellular localization of Nrf2 was determined using immunostaining method. (b) HSF cells were cotransfected with pGL3-ARE and treated with various concentrations of ZER (2-8 *μ*M for 2 h) to measure the percentage of ARE promoter activity. Data was presented as fold over increase in the percentage of ARE promoter activity. (c, d) The effect of ZER treatment (8 *μ*M) on the expression of total Nrf2 and antioxidant proteins (HO-1 and *γ*-GCLC) at different time points (0, 1, 2, 4, 8, 12, 16, or 24 h) was measured using western blot method against *β*-actin as an internal control. Results from three or more experiments were presented as mean ± SD, and the statistical significance was considered as ∗*p* < 0.05, ∗∗*p* < 0.01, ∗∗∗*p* < 0.001 compared to untreated control cells.

HO-1 and *γ*-GCLC antioxidant gene induction was the primary event associated with the scavenging of ROS to protect the cells from oxidative insults. This effect was mediated via the nuclear translocation of Nrf2 [49, 50]. From this, we first tested the ZER-induced nuclear localization of Nrf2 in HSF cells through immunofluorescence staining method.  Figure 5(a) shows that compared to the untreated cells, 8 *μ*M ZER dramatically increased the Nrf2 fluorescence intensity indicating an increased Nrf2 nuclear localization in the presence of ZER. Using ARE-harboring luciferase reporter system, we tested the activity of ARE involved in Nrf2 transcriptional activation due to ZER. Our experimental data showed that ZER treatment (2-8 *μ*M) significantly upregulated the ARE-promoter activity in a dose-dependent manner. Therefore, we concluded that ZER promoted ARE activity and nuclear translocation of Nrf2 in HSF cells (Figure 5(b)). Later, we tested the ZER-mediated time-dependent expressions of *γ*-GCLC and HO-1 proteins in HSF cells. As shown in Figures 5(c) and 5(d), 8 *μ*M ZER treatment significantly upregulated the expression of HO-1 protein levels from 4 to 24 h time points, whereas *γ*-GCLC levels were upregulated up to 4 h time point and then gradually decreased till 24 h (Figures 5(c) and 5(d)). This data confirmed that ZER was mediating the nuclear localization and transcriptional activation of Nrf2 that further promotes the induction of HO-1 and *γ*-GCLC proteins in HSF cells.

### 3.8. ZER-Induced Nrf2 Activation Was Mediating via Various Signal Transduction Pathways

**Figure 6:**
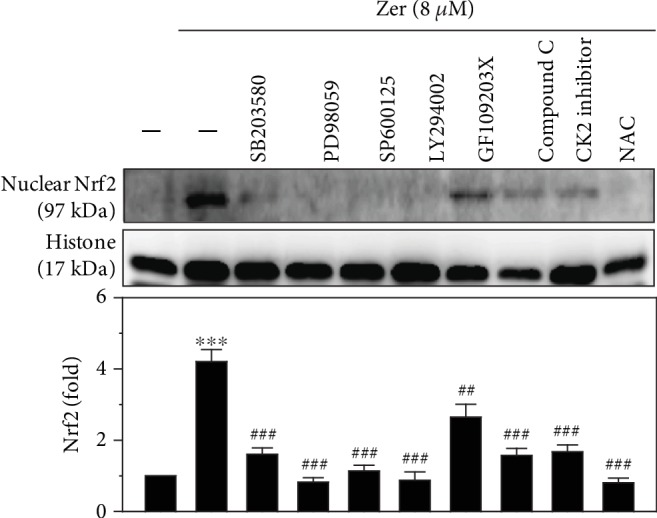
Role of signal transduction pathways mediating the activation of nuclear Nrf2 in ZER-pretreated HSF cells. Cells were pretreated with pharmacological inhibitors for MAPK p38 (SB203580, 20 *μ*M), ERK (PD98059, 30 *μ*M), JNK (SP600125, 25 *μ*M), PI3K/AKT (LY294002, 30 *μ*M), PKC (GF109203X, 2.5 *μ*M), AMPK inhibitor (compound C, 10 *μ*M), casein kinase II inhibitor (CKII inhibitor, 20 *μ*M), or ROS inhibitor (NAC, 1 mM) for 30 min followed by ZER treatment (8 *μ*M) for 2 h. Cells were harvested and nuclear protein fraction was analyzed using western blot method. Histone was used as an internal protein control. Results were presented as mean ± SD of three assays. ∗∗∗*p* < 0.001 compared to untreated control cells; ^##^*p* < 0.01, ^###^*p* < 0.001 compared to ZER alone-treated cells.

Previous studies have reported that Nrf2 activation and regulation were mediated via various signaling pathways [42, 50]. In this study, we pretreated the HSF cells with various pharmacological inhibitors against ERK (PD98059, 30 *μ*M), PI3K/AKT (LY294002, 30 *μ*M), JNK (SP600125, 25 *μ*M), p38 MAPK (SB203580, 20 *μ*M), PKC (GF109203X, 2.5 *μ*M), casein kinase II (CKII inhibitor, 20 *μ*M), AMPK (Compound C, 10 *μ*M), and ROS (NAC, 1 mM) for 30 min, followed by 8 *μ*M ZER treatment for 2 h. Cells were harvested, and western blot was performed to check the signaling pathways involved in ZER-induced Nrf2 activation and regulation in HSF cells. Our data showed that ZER substantially upregulated the expression of Nrf2 in ZER alone-treated cells (Figure 6). However, this effect was statistically downregulated in the presence of all the abovementioned pharmacological inhibitors inferring these signaling pathways are mediating the ZER-induced Nrf2 activation and regulation in HSF cells (Figure 6).

### 3.9. Antiphotoaging Effect of ZER Was Diminished due to Nrf2 Knockdown

**Figure 7:**
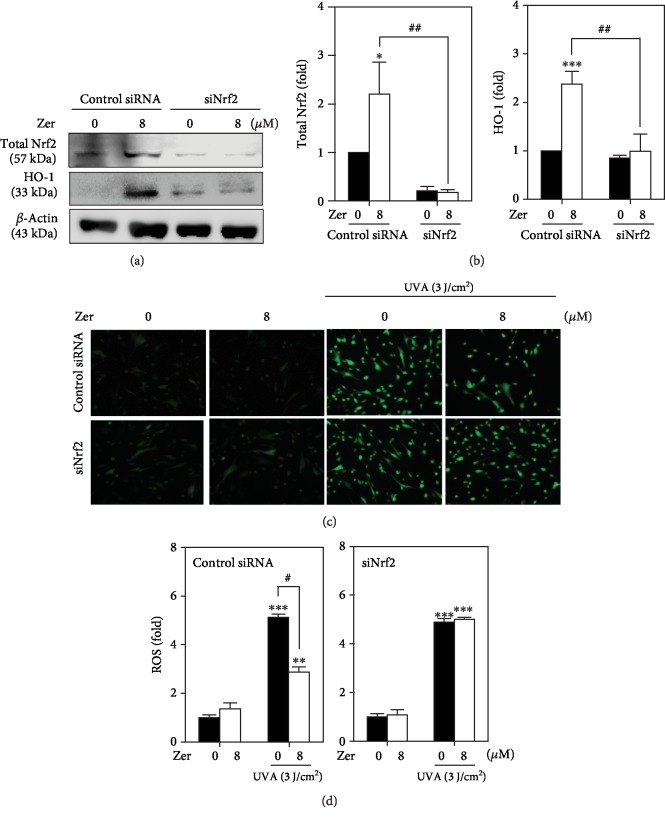
ZER-mediated protective effect against UVA radiation was attenuated in Nrf2 knockdown HSF cells. (a, b) Cells were transfected with specific siRNA against Nrf2 or a nonsilencing control, followed by treatment with ZER (8 *μ*M, 2 h), and the expression patterns of total Nrf2 and HO-1 proteins in both Nrf2-transfected and nonsilencing control were measured using western blot method. (c, d) In ZER-pretreated (8 *μ*M, 24 h) and UVA-exposed HSF cells, the effect of Nrf2 silencing on intracellular ROS accumulation was measured using DCFH_2_-DA (10 *μ*M) fluorescence staining method. Data were presented as fold change of ROS in nonsilencing control and Nrf2 knockdown cells. Results were presented as mean ± SD of three or more assays. ∗*p* < 0.05, ∗∗*p* < 0.01, ∗∗∗*p* < 0.001 compared to untreated control cells. ^#^*p* < 0.05, ^##^*p* < 0.01, compared to ZER-treated cells.

To further confirm that Nrf2 activation is essential for ZER to exhibit its dermatoprotective properties in UVA-irradiated cells, we performed Nrf2 knockdown studies and measured ROS levels as well as the expressions of total Nrf2 and HO-1 protein levels in HSF cells. siRNA specific for Nrf2 or control was transfected to HSF cells and exposed to UVA radiation in the presence or absence of ZER. Blunted Nrf2 levels confirmed the successful knockdown of Nrf2 in transfected cells. ZER-induced total Nrf2 and HO-1 expressions were dramatically decreased in Nrf2-siRNA-transfected cells following UVA exposure (Figures 7(a) and 7(b)). Interestingly, UVA-induced ROS accumulation in Nrf2 knockdown cells remains high but was downregulated in the control cells even after ZER treatment (Figures 7(c) and 7(d)). Our results confirmed that Nrf2 knockdown accumulated the UVA-induced ROS levels leading to dysregulation in cellular homeostasis in HSF cells.

## 4. Discussion

There is a dramatic increase in the incidence of UVA radiation-induced photobiological damage to skin cells. UVA penetrates deep into the skin and damages the dermal compartment, which leads to wrinkles, photoaging, and skin cancer [2]. With the rapid progress in cosmetic health and quality of life, use of safe and highly effective phytochemicals that protect the skin from these deleterious effects has become a need of the hour [51, 52]. Several studies had reported that zerumbone (ZER), a monocyclic sesquiterpene compound (Figure 1(a)) extracted from the rhizomes of *Zingiber zerumbet*, possesses remarkable antimicrobial [53], antihyperglycemic [54], antiallergic [55], anti-inflammatory [56], anticancer [57, 58], and antihypercholesterolemic and antioxidant [59–61] properties. In this study, we tested the antioxidant, antisenescence, and cell proliferative properties of ZER in UVA-irradiated HSF cells and the molecular mechanisms that underlie these properties.

UVA stress is known to cause alterations in the dermal matrix and impairs fibroblast homeostasis leading to wrinkles, coarseness, and laxation in the human skin [62]. Quan et al. reported that MMP-1 is a major collagenolytic enzyme that degrades the collagen present dermal compartment due to radiation insult. Therefore, MMP-1 was considered to be an important “photoaging marker” in skin cells [45, 63, 64]. Here, we tested the effect of ZER's anticollagenolytic activity in UVA-irradiated HSF cells. Our data showed that ZER pretreatment attenuated the UVA-induced MMP-1 expression and reversed the degradation of its target protein collagen III (Figures 1(c) and 1(d)). Additionally, cell death resulted from UVA exposure was suppressed due to ZER pretreatment leading to an increase in the percentage of viable HSF cells, which allows us to conclude that ZER possesses cell proliferation activity (Figure 1(b)).

UVA radiation triggers the accumulation of intracellular ROS levels in different cell types [32, 41, 65]. This increase in ROS levels led to cellular senescence in skin cells and is said to be a precursor cause for premature skin aging. SA-*β*-gal is a key biomarker associated with this cellular senescence activity [41, 44, 66]. In this study, we first measured the accumulation of intracellular ROS levels followed by SA-*β*-gal activity in UVA-irradiated HSF cells. Our data showed approximately 3.5-fold increase in intracellular ROS levels and 55% of SA-*β*-gal-positive HSF cells due to UVA stress. However, this effect was attenuated in the presence of ZER. All these observations deduced the antioxidant and antisenescence abilities of this compound in skin cells (Figures 2(a)–2(c)).

Protein phosphorylation is a key mechanism involved in regulating the activity of transcription factors in both upregulated or downregulated manners [67]. Pillai et al. have reported that activation of transcription factor AP-1 was important in UVA-induced ROS accumulation in premature skin aging [47, 48]. Our protein expression data suggested that UVA radiation alone dramatically increased the phosphorylations of c-Fos and c-Jun proteins in HSF cells. But, this effect was suppressed in the presence of ZER in a dose-dependent pattern with maximum effect was observed at 8 *μ*M ZER concentration. Thus, ZER controls the UVA-induced nuclear accumulation of p-c-Fos and p-c-Jun in HSF cells (Figures 3(a)–3(d)).

Activation of Nrf2 antioxidant response element (Nrf2-ARE) is a well-known signaling pathway mediating the expression of various cytoprotective proteins that helps in detoxifying the cellular reactive oxidants and electrophile stimuli. The activity of Nrf2 is controlled, in part, by its cytosolic-associated protein Keap-1. Sequestration of Keap-1 from Nrf2-Keap-1 complex determines cellular homeostasis [49]. Results from our study have shown that ZER treatment (8 *μ*M) mediated the translocation of cytosolic Nrf2 to nucleus that was first observed at 0.5 h time point and was gradually increased up to 4 h time point. It is noteworthy that there was a consistent decrease in cytosolic Nrf2 implicating that ZER pretreatment activated the dissociation of Nrf2-Keap-1 complex in the cytosol and favoring the nuclear translocalization of Nrf2 to mediate ZER's cytoprotective downstream effects (Figures 4(a)–4(f)).

HO-1 and *γ*-GCLC antioxidant gene induction was the primary event associated with scavenging of ROS to protect the cells from oxidative insult. This effect was mediated via the nuclear translocation of Nrf2 [50]. Nrf2 binds to cis-acting AREs present in the promoter region of antioxidant and detoxifying phase II encoding genes that lead to the expression of their respective proteins. Nrf2 functions as a master regulator for cellular responses to oxidant injuries [68]. In support of this, our experimental data also demonstrated that 8 *μ*M ZER substantially increased the nuclear translocation of Nrf2 that was consistent with significant upregulation in ARE promoter activity in HSF cells (Figures 5(a) and 5(b)). These observations determined the expression patters of *γ*-GCLC and HO-1 proteins in HSF cells. Our western blot results showed that both these proteins were expressed in a time-dependent manner (Figures 5(c) and 5(d)). Therefore, we conclude that ZER was mediating the nuclear localization and transcriptional activation of Nrf2 to induce the expression of HO-1 and *γ*-GCLC proteins in HSF cells. We further conducted Nrf2 knockdown studies and determined that Nrf2 activation was essential for ZER to mediate the expression of antioxidant proteins. Figures 7(a)–(d) show that ZER-induced total Nrf2 and HO-1 expressions were dramatically decreased in Nrf2-siRNA-transfected cells after UVA exposure. Most importantly, UVA-induced ROS accumulation in Nrf2 knockdown cells remains high but was substantially inhibited in the control cells after ZER treatment. This data elucidated that ZER was not able to suppress the UVA-induced ROS generation in the absence of Nrf2.

Muthusamy and Piva reported that MAPKs play a pivotal role in transmitting the environmental stress signals, such as UVA radiation, to the cytoplasm and to the nucleus [69]. All three pathways of MAPKs are important and interconnected to stimulate transcription factor AP-1 [70]. The phosphorylation of specific threonines and tyrosines also initiates the translocation of MAKs into the nucleus that results in the activation of c-Jun and c-Fos [71]. ERK is critical to drive c-Fos, whereas activation of JNK and p38 is important for c-Jun stabilization [71]. We used specific pharmacological inhibitors to evaluate the role of various signal transduction pathways involved in ZER-mediated Nrf2 activation and nuclear translocation. Data from our studies clearly showed that ZER-induced Nrf2 activation and translocation were mediated via ERK, PI3K/AKT, JNK, p38, PKC, CK-II, AMPK, and ROS pathways (Figure 6).

## 5. Conclusions

In summary, this study delineated the molecular mechanisms underlying the ZER-induced activation of Nrf2 and its subsequent downstream protective effects in UVA-stressed HSF cells. ZER increased cell viability and suppressed the cellular senescence activity in HSF cells. In addition to this, ZER suppressed the UVA-induced expression of collagenolytic activity of MMP-1 and prevented the cells from undergoing matrix degradation. Moreover, ZER favors nuclear translocation and the activation of Nrf2 to mediate the induction of HO-1 and *γ*-GCLC genes. Interestingly, Nrf2 knockdown was not able to show the expression of these proteins. Moreover, ZER-induced Nrf2 activation was mediated via various signal transduction pathways. Our findings concluded that ZER may consider as a nutritional supplement that may be used in the formulation of natural skin care products to treat and help skin cells from radiation-induced damages and premature skin aging.

## Data Availability

The data used to support the findings of this study are included within the article.
